# Mechanical properties of 3D voxel-printed materials for cardiovascular tissue imitation

**DOI:** 10.3389/fbioe.2025.1569553

**Published:** 2025-05-30

**Authors:** Joël Illi, Manuel Bergamin, Marc Ilic, Anselm W. Stark, Stefan Bracher, Martin Hofmann, Juergen Burger, Isaac Shiri, Andreas Haeberlin, Christoph Gräni

**Affiliations:** ^1^ Department of Cardiology, Inselspital, Bern University Hospital, University of Bern, Bern, Switzerland; ^2^ ARTORG Center for Biomedical Engineering Research, University of Bern, Bern, Switzerland; ^3^ School of Biomedical and Precision Engineering, University of Bern, Bern, Switzerland; ^4^ Translational Imaging Center, Sitem Center, University of Bern, Bern, Switzerland

**Keywords:** patient-specific phantoms, 3D-printing, additive manufacturing, artificial cardiovascular tissue, non-linear material mechanics, voxel-printing, cardiovascular phantoms, uniaxial tensile test

## Abstract

**Background:**

Cardiovascular patient-specific phantoms can improve patient care through testing and simulation. However, materials like silicone and 3D-printing polymers differ mechanically from biological tissues. Agilus30 Clear, the primary material for 3D-printed phantoms, is much stiffer, nearly isotropic, and lacks strain-hardening behavior. To overcome these challenges, a novel 3D voxel-printing approach may provide an effective solution.

**Methods/aim:**

This study aimed to explore the applicability of 3D voxel printing, assess how different parameters (strand structure, density, and orientation) affect mechanical properties, and compare them to established phantom materials and porcine cardiovascular tissues. Progressive uniaxial cyclic tension tests were performed across nine stages, varying strain rates and target strain levels, with elastic modulus calculated for comparison. The goal was to stepwise assess whether the overall material stiffness can be reduced, achieving anisotropy and replicating strain-hardening behavior.

**Results:**

In the first step, varying the strand density, the tested samples showed a 0%–60% strain modulus of elasticity of 0.215–0.278 N/mm^2^, representing a 4–5-fold reduction in elastic modulus compared to that of the base material, Agilus30 Clear. In the second step, varying the orientation of the structures had a significant influence on the elastic modulus, which was measured. The 0%–60% modulus of elasticity decreased to 0.161–0.192 N/mm^2^, displaying anisotropic material behavior. In the third step, two strand structures specifically designed to mimic fiber recruitment were tested. These resulted in slightly flatter (more linear) stress–strain curves compared to the non-linear strain-softening behavior observed in Agilus30 Clear. However, they still fell short of replicating the desired non-linear strain-hardening behavior characteristic of fiber recruitment in cardiovascular tissues.

**Conclusion:**

The novel 3D voxel-printing material approach resulted in reduced elastic modulus, anisotropic behavior, and strain-hardening properties, providing a much closer representation of the mechanical behavior of porcine cardiovascular tissues compared to other available phantom materials. However, there is still significant potential for optimization through further exploration of fiber recruitment replication.

## 1 Introduction

**FIGURE 1:**
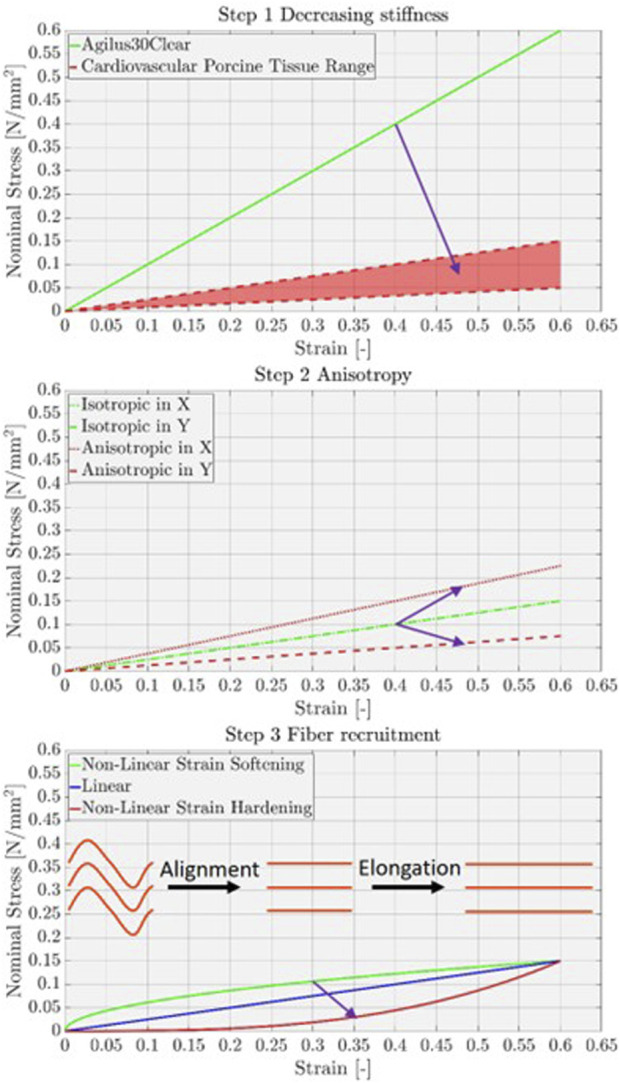
Graphical representation of the three desired optimization steps toward more physiological 3D printing materials. Step 1: decreasing slope to the porcine cardiovascular tissue range. Step 2: changing material behavior from isotropic (independent from the loading axis direction) to anisotropic (dependent on the loading axis direction). Step 3: changing material behavior from non-linear strain softening (slope decreases with increasing strain) to strain hardening (slope increases with increasing strain). Visualization of the strand recruitment. The recruitment point marks the transition from the strands aligning in the loading direction to the elongation of the strands.

Cardiovascular phantoms serve diverse purposes, including education, clinical training, pre-operative planning, and hemodynamic testing. They are further increasingly used in device testing, development, and validation and are being explored as physical artificial twins for pre-clinical evaluations (Illi et al., 2024). With their increasing clinical relevance, the mechanical properties of phantom materials are becoming a critical factor. Additionally, the demand for patient-specific designs and the shorter production times required in clinical settings make directly additively manufactured (AM) phantoms the preferred choice (Illi et al., 2022; Bernhard et al., 2022). Therefore, there is a need for 3D-printing materials and processes capable of producing phantoms that more accurately mimic physiological mechanical behavior (Illi et al., 2023; Scanlan et al., 2018; Yoo et al., 2017; El Sabbagh et al., 2018; Kim et al., 2021; Park and Kim, 2022; Wang et al., 2016). The primary limitations of currently used phantom materials can be categorized into three key issues—stiffness, isotropy, and stress–strain behavior—ranked in order of decreasing importance. To address these challenges, optimizing the commonly used Agilus30 material (Illi et al., 2022; Bernhard et al., 2022) toward achieving a mechanical behavior that more closely mimics soft or cardiovascular tissue is necessary (Figure 1). One important focus is reducing the overall stiffness of the material by lowering the modulus of elasticity, which represents the ratio between internal material stress and applied elongation, or the slope of the stress–strain curve (Emig et al., 2021). Agilus30 and other commonly used materials exhibit a non-linear strain-softening behavior in the strain range between 0% and 30% (Wang et al., 2016), with a slope that is five to seven times higher than that of porcine cardiovascular tissues, depending on the strain range (Voigt and Cvijic, 2019). Additionally, soft tissues are known to exhibit anisotropic behavior, indicating that the resulting forces vary depending on the direction of the applied deformation due to fiber distribution and orientation (Sommer et al., 2015; Nemavhola, 2021). To replicate this behavior, an oriented structure is required as Agilus30, like silicone cast, behaves in a primarily isotropic manner (Illi et al., 2023). Furthermore, the slope of stress–strain curves for soft tissues is non-linear and demonstrates strain-hardening behavior due to fiber recruitment (Zhalmuratova et al., 2019). This characteristic requires a structure that can mimic this non-linear behavior. By leveraging multi-material voxel printing based on PolyJet printing technology, we aimed to develop metamaterials specifically designed to mimic the mechanical properties of soft tissues.

Therefore, the goal of this study is to test the feasibility of this approach, explore the influence of material parameters on these new materials in a stepwise manner, and compare their mechanical properties with existing data on porcine cardiovascular tissues and cardiovascular phantom materials, while maintaining consistency in setup, geometry, and protocols with previous measurements, across three steps to address the three aforementioned limitations.

## 2 Materials and methods

### 2.1 Samples

Based on mechanical testing findings from previous research (Illi et al., 2022; Bernhard et al., 2022), including our study (Illi et al., 2023), we decided to focus on the direct AM approach using the PolyJet technology and voxel printing. This approach leverages the multi-material capabilities of the technology, including the use of compatible materials and its voxel-based printing process. We used the voxel-printing approach to create different structures addressing the three outlined steps. Table 1 provides an overview of all tested structures, including their parameters and the number of samples manufactured and tested.

**TABLE 1:** Tested voxel-printing structures.

Structure	Organization	Orientation [°]	Recruitment point [%]	Strand density [–]	Gap [voxel]	Survival
L-00°-00%-3-0	Linear	0	0	3	0	5/5
L-00°-00%-4-0	Linear	0	0	4	0	5/5
L-00°-00%-5-0	Linear	0	0	5	0	4/5
L-30°-00%-3-2	Linear	±30	0	3	2	5/5
L-30°-00%-4-0	Linear	±30	0	4	0	5/5
L-60°-00%-3-2	Linear	±60	0	3	2	0/5
L-60°-00%-4-0	Linear	±60	0	4	0	5/5
S-00°-17%-3-2	Sinusoidal	0	17	3	2	0/5
S-00°-17%-4-0	Sinusoidal	0	17	4	0	4/5
S-00°-30%-3-2	Sinusoidal	0	30	3	2	0/5
S-00°-30%-4-0	Sinusoidal	0	30	4	0	5/5
S-30°-17%-3-2	Sinusoidal	±30	17	3	2	0/5
S-30°-17%-4-0	Sinusoidal	±30	17	4	0	5/5
S-30°-30%-3-2	Sinusoidal	±30	30	3	2	0/5
S-30°-30%-4-0	Sinusoidal	±30	30	4	0	5/5
P-00°-00%-0-0	Pure	0°	0	0	0	5/5
				Helix diameter [–]	Helix gap [–]	
H-00°-20%-8-1	Helical	0	20	8	1	5/5
H-00°-40%-8-1	Helical	0	40	8	1	5/5
H-30°-20%-8-1	Helical	±30	20	8	1	5/5
H-30°-40%-8-1	Helical	±30	40	8	1	5/5

Table of the tested voxel-printing structures with characterizing parameters and number of samples that survived the 10 fast cycles to 60% strain without rupturing. P-00°-00%-0-0 represents pure Agilus30 Clear with the results from our previous study for the sample Agilus30 Clear-Along.

#### 2.1.1 Materials

For the voxel-printing approach, the PolyJet printing technology from Stratasys Additive Manufacturing (Rehovot, Israel) was used, along with their proprietary PolyJet materials (i.e., Agilus30 Clear, VeroBlackPlus, and SUP706B) (Illi et al., 2023).

#### 2.1.2 Geometry

**FIGURE 2:**
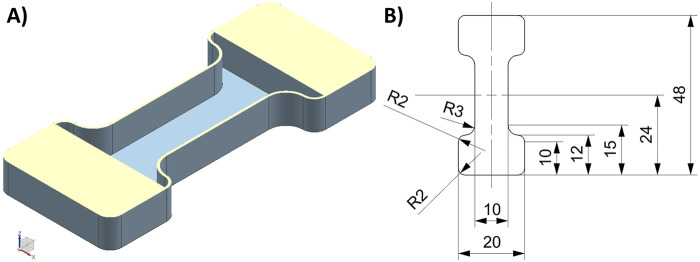
Designed dumbbell sample geometry. **(A)** Isometric view of the horizontal section of the Agilus30 Clear envelope/shell. The reinforcement of the clamping portion was 10 mm long, and the wall thickness of the remaining shell was 0.4 mm. The sample thickness was 10 mm for these tests. **(B)** Dimensioned drawing of the sample geometry. The gage length was 28 mm. Dimensions are shown in mm. R, radius.

The sample geometry was a custom design based on standardized tension testing geometries (dogbone/dumbbell), as reported previously (Illi et al., 2023) (Figure 2). The samples consisted of a pure Agilus30 Clear envelope with a thickness of 0.4 mm, encapsulating the voxel-printed structures within. The envelope protects the delicate structures during support removal and ensures water tightness, which is essential for hemodynamic testing applications, such as in a flow-loop setup (Illi et al., 2024). We designed the lower part of the clamping portion of the samples to be pure Agilus30 Clear to ensure load transformation and sample integrity.

#### 2.1.3 Sample design and material parameters

The envelope and clamping portion were drawn conventionally using CAD in NX 1884 (Siemens Digital Industries Software, Plano, United States) in the voxel domain. The internal filling of the envelope, including the structures, was calculated using MATLAB (The MathWorks, Inc., Massachusetts, United States) and constructed based on parameters. In addition to the organization of the strands (linear, sinusoidal, and helical), various parameters such as material composition, strand density, layer gap, layer orientation, amplitude, wavelength, pitch, and diameter were used to create the different structures.

**FIGURE 3:**
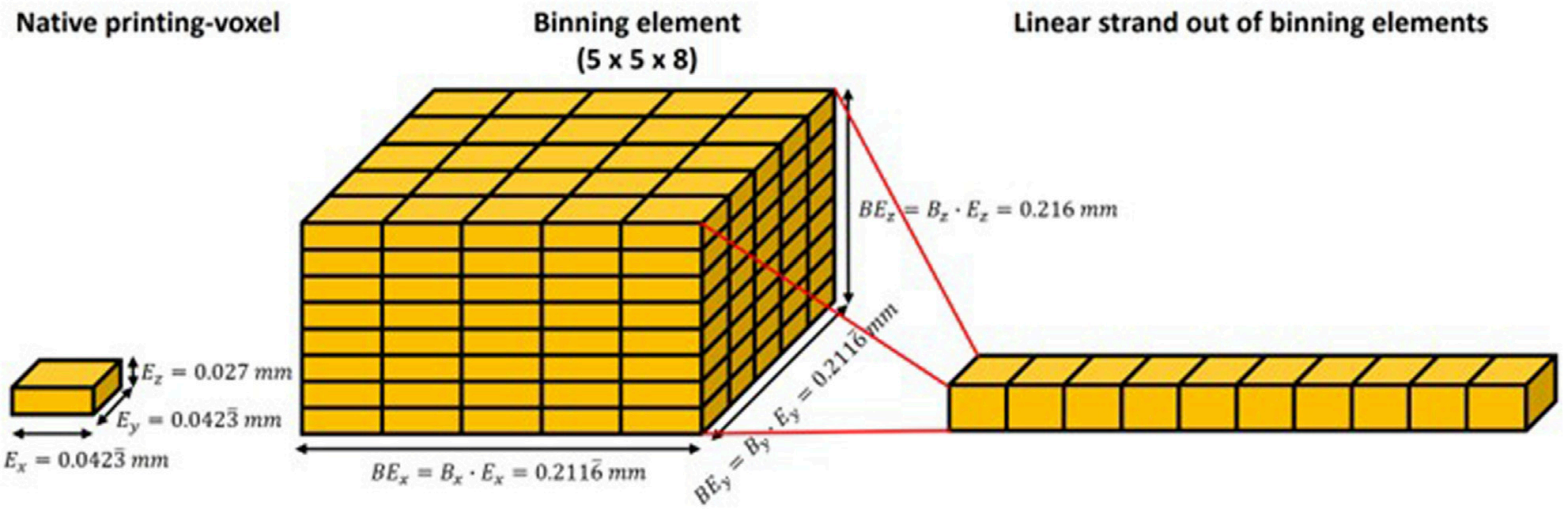
Depiction and dimensions of the printing voxels, binning, and strands. Depiction of the constructive process from native printing voxels to binning elements to strands to create the tested structures. The dimensions of the native printing voxel correspond with the printer’s listed native resolution for printing with Agilus30.

The printer has a native resolution of 600 dpi on the X- and Y-axes, which results in a planar resolution of 42.3 × 42.3 μm, and the Z-axis resolution is 27 μm. To improve the fidelity of the printing process and reduce the computational cost, we used cubic binning elements with 5 × 5 × 8 voxels and a dimension of 212 × 212 × 216 μm to create the voxel-printing structures (Figure 3).

**FIGURE 4:**
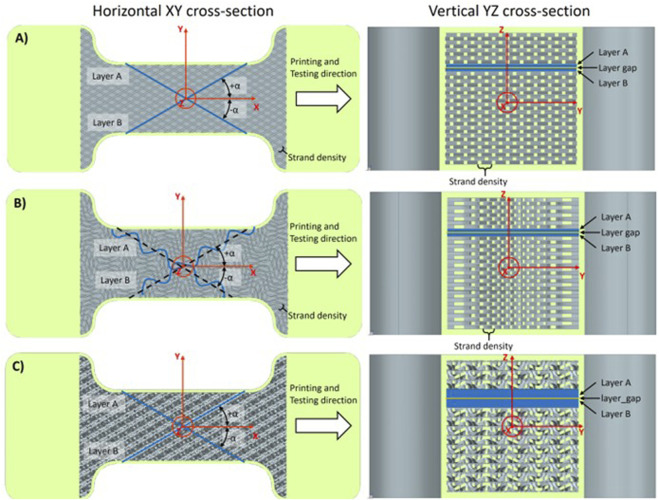
Depiction of the voxel-printing structures. Horizontal (XY) and vertical (YZ) cross-sections of the voxel-printing structures in CAD with basic parameters and cutting surfaces (light green). **(A)** Linear structure with ±30° orientation and a layer gap. **(B)** Sinusoidal structure with ±30° orientation and a layer gap. **(C)** Helical structure (paired double helices with right- and left-handed twists) with ±30° orientation and a layer gap.

The following three steps were defined to meet the preferred requirements shown in Figure 1. The structures are built of repeating layers A and B, separated by a layer gap. The organization and strand density in the A layer were identical to those of the B layer. The direction of the strands in layer A was oriented at a clockwise (+) angle to the loading axis, while in layer B, the strands were oriented anti-clockwise (−), maintaining symmetry with respect to this axis. For comparability reasons, the cross-sectional material density of Agilus is kept constant, between 50% and 30%, including the envelope for all samples. For example, when the layer gap is increased, the number of strands per layer is also increased to maintain the same or similar cross-sectional material density. The strand density value is inversely proportional to the number of strands per layer (one in X voxels is Agilus/material, while X-1 voxels are void/support), and thus to the material density. Figure 4 shows the three base organizations, with the layer build-up and the mentioned parameters. For more details and quantitative information on the structures, organization, and parameters, refer to the Annex.

##### 2.1.3.1 First step—stiffness

In the first step, we used a linear organization with strands of binning elements aligned along the applied loading direction (0°). Strand density was varied to create different structures, with different stiffnesses. With linear strands, there is no recruitment point (0% strain) as the strands are immediately engaged under load. Due to the 0° orientation, there is no layer gap needed to avoid cross-linking between the strands.

To investigate the influence of the strand density parameter, we selected values of 3, 4, and 5. Based on our pre-trials, this should render results slightly above the upper boundary of the physiological range from our previous investigations (Illi et al., 2023) and within it.

##### 2.1.3.2 Second step—anisotropy

In the second step, the same linear organization as in the first step was used, with strand orientation varied per layer (A: clockwise/+ and B: counterclockwise/−) to generate distinct anisotropic structures. For orientation, the tensile direction was defined as the 0° reference. An angle of 30° was chosen to reflect the predominant fiber orientation in large vessels, which is densest between 30° and 40° (Flamini et al., 2013; Schriefl et al., 2012). Additionally, 60° was included to represent the orthogonal orientation, corresponding to the axial versus circumferential directions of the samples. This change in orientation per layer leads to additional cross-linking of the strands between the layers, where they overlap, which stiffens the structures. To investigate and control this effect, we implemented a parameter for a layer gap (void space between the layers) to be able to turn it on and off. The resulting decrease in material density due to the layer gap was compensated by decreasing the strand density value to keep the overall material density comparable. In order to avoid significantly altering the material density, the smallest layer gap that could be produced reliably was used. This gap corresponded to two voxels. Likewise, as in the first step, there was no recruitment point (0%), and the strands were loaded immediately.

##### 2.1.3.3 Third step—strain hardening

In the third step, to mimic the non-linear strain-hardening behavior of soft tissue, two distinct strand organizations, sinusoidal and helical, were implemented to replicate this behavior.

The sinusoidal structures consist of sinusoidally arranged binning elements, forming the strands. The ratio between the path length of the strand and the wavelength defines the recruitment point in % strain, where the strands are aligned in the loading direction and start to elongate (Figure 1, Step 3).

Due to the limitations of the discrete voxel domain, where dimensions must be multiples of voxel size, and the decision to vary only one parameter of the path length, the resulting strain values for the recruitment points are also discrete. Based on the findings from our previous study, strain levels of 17% and 30% deformation were selected (Illi et al., 2023).

For the helical organization, right-twisted double helices composed of binning elements were alternated with left-twisted double helices in each layer to prevent the transformation of axial elongation into rotation. Furthermore, layers A and B were mirrored so that each left-twisted double helix was surrounded by right-twisted ones and *vice versa*. Furthermore, those double helices were separated by the helix gap to prevent cross-linking. The recruitment point for this organization is defined through the helix pitch and diameter and also denoted in % strain.

As with the sinusoidal structures, the recruitment points for the helical structures were limited to discrete strain values. Anticipating that the separated strands would come into contact and influence the mechanical response before full alignment, we selected recruitment points at higher strains, of 20% and 40%, than those used in the sinusoidal designs.

#### 2.1.4 Manufacturing of the samples

**FIGURE 5:**
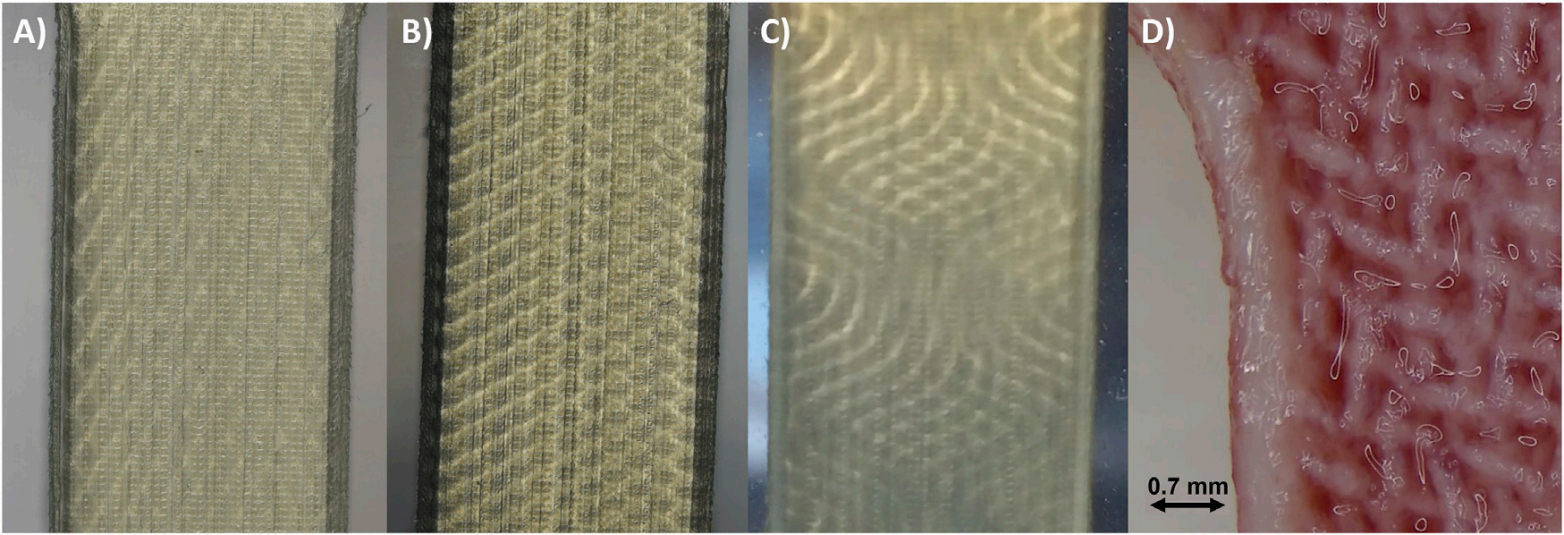
Detailed view of the various voxel-printing structures. **(A)** Image of a sample with a linear structure with ±30° orientation and **(B)** ±60° orientation. **(C)** Sinusoidal structure with ±30° orientation. **(D)** Microscopic image of a dyed sample with a helical structure with ±30° orientation. Reference scale (red) corresponds to 0.7 mm.

All samples were sliced using GrabCAD Print V1.75, with model settings set to Matte Finish and support strength set to light. Post-printing, the support material was mechanically removed using a brush under running water. Figures 5A–D presents three images of tested samples with linear and sinusoidal organizations, along with a microscopic image of a dissected and dyed sample featuring a helical organization.

### 2.2 Mechanical testing

**FIGURE 6:**
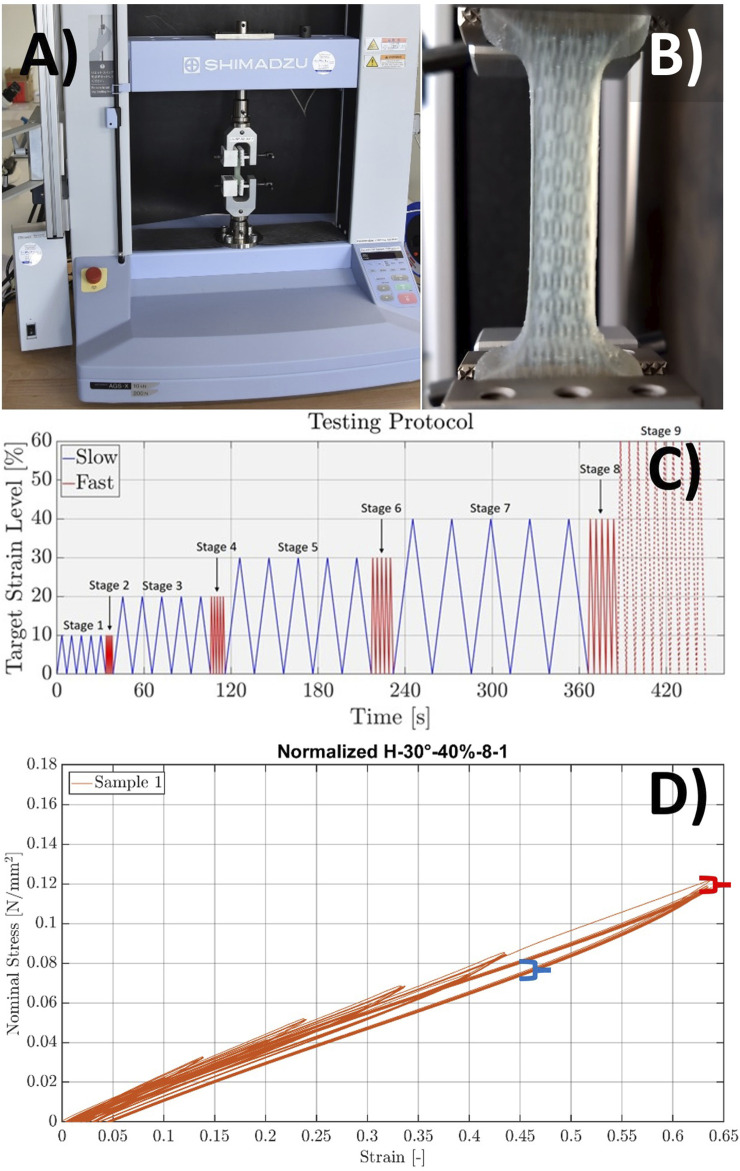
Mechanical testing setup and data processing. **(A)** Uniaxial tension testing setup with the clamped pure Agilus30 Clear sample. **(B)** Voxel-printing sample during testing at a peak strain of 60%. **(C)** Visual representation of the testing protocol in the form of a time–strain plot, with the slow cycles shown in blue, fast cycles in red, and the last stage with a dotted red line. **(D)** Raw data on one sample after conversion from force–displacement to stress–strain, with marked hysteresis represented in blue and adaptation effect in red.

The material testing was performed on a uniaxial tensile testing machine (Autograph AGS-X 200N, Shimadzu Corporation, Kyoto, Japan). Its proprietary software (Trapezium X Materials Testing Software v1.5.3, Shimadzu Corporation, Kyoto, Japan) was used to record the forces and deformations at a sampling rate of 100 Hz. In addition to the proprietary 200N load cell, standard vice grips and gripping plates with a pyramidal structure were used (Figure 6A). The variation in sample weight and compressive force was compensated by calibration and a compensation routine (0-hold). The initialization of the protocol consisted of a preloading routine, with a preload of 0.05 N at a deformation speed of 2 mm/min. The protocol consisted of five cycles at a lower deformation and relaxation speed (strain rate) of 50 mm/min (∼3%/s), denoted slow, followed by five cycles at higher speeds (strain rates) of 500 mm/min (∼30%/s) deformation and 250 mm/min (∼15%/s) relaxation, denoted fast. This was repeated for the target strain levels of 10%, 20%, 30%, and 40% at both deformation speeds, creating the first eight stages. The last, ninth stage consisted of 10 cycles to 60% strain (Figure 6B) with the fast rates, following the same protocol as our previous study (Illi et al., 2023). The first eight stages were added to gain more information about the material behavior and account for possible premature ruptures while still ensuring comparability to our reference data. An explanatory table and plot of the testing protocol can be found in Table 2 and Figure 6C.

**TABLE 2:** Mechanical testing protocol.

Cycle	Stage	Deformation speed and strain rate (deformation and relaxation)	Target deformation
1	1	Slow 50 and 50 mm/min 3% and 3%/s	10%
2			
3			
4			
**5**			
6	2	Fast 500 and 250 mm/min 30% and 15%/s	
7			
8			
9			
**10**			
11	3	Slow 50 and 50 mm/min 3% and 3%/s	20%
12			
13			
14			
**15**			
16	4	Fast 500 and 250 mm/min 30% and 15%/s	
17			
18			
19			
**20**			
21	5	Slow 50 and 50 mm/min 3% and 3%/s	30%
22			
23			
24			
**25**			
26	6	Fast 500 and 250 mm/min 30% and 15%/s	
27			
28			
29			
**30**			
31	7	Slow 50 and 50 mm/min 3% and 3%/s	40%
32			
33			
34			
**35**			
36	8	Fast 500 and 250 mm/min 30% and 15%/s	
37			
38			
39			
**40**			
41	9	Fast 500 and 250 mm/min 30% and 15%/s	60%
42			
43			
44			
45			
46			
47			
48			
49			
**50**			

List of the individual cycles of the testing protocol, with corresponding stage, deformation speed, and target deformation. The evaluation cycles for the data evaluation are marked in bold.

### 2.3 Data evaluation

The raw data were processed using MATLAB (The MathWorks, Inc., Massachusetts, United States). The raw force–displacement data were converted into nominal or engineering stress by dividing the force by the initial cross-sectional area. Strain was defined as the ratio of the displacement and the initial gauge length. Due to the minimal deviation from the designed values, the designed thickness and width were used to calculate the initial cross-sectional area.

The converted data were then separated into 50 individual loading and unloading cycles. The separation into loading and unloading cycles is necessary to account for hysteresis, the difference in path between unloading and loading (Figure 6D). For each stage (target strain level and strain rate), only the last cycles were processed for evaluation to account for the adaptation effect. It describes the asymptotic reduction in stiffness between subsequent cycles during cyclic testing of soft elastic materials (Lokshin and Lanir, 2009) (Figure 6D). Evaluation of the first cycles or averaging over the first cycles would lead to an overestimation of the resulting forces. Thus, the initial cycles can be considered preconditioning, where a stabilization between cycles 2 and 4 (Sommer et al., 2015; Peña et al., 2009; Dokos et al., 2002; Li et al., 2022) is observed, leading to cycles 5, 10, 15, 20, 25, 30, 40, and 50 being used for further evaluation. Those results were then averaged over all five samples with the same structure (organization and parameters) to create the material curves. Figure 6D shows an example of a sample curve with all cycles after conversion into a stress–strain curve. To quantitatively compare the structures to each other and the reference materials, the incremental modulus of elasticity (slope of the stress–strain curves) of the loading cycles was calculated for the following four strain ranges, namely, 0%–10%, 10%–20%, 20%–30%, and 30%–40%, for both strain rates. For the 40%–60% and 0%–60% strain ranges, only the fast strain rate for each sample was calculated. Finally, the median and interquartile ranges (IQRs) of those values for each structure were calculated.

## 3 Results

All samples demonstrated lower stress levels during unloading than those during loading in all cycles. A measurable reduction in peak stress was observed in the first three cycles for each sample, with a decrease in magnitude from cycle to cycle. This pattern was repeatedly consistent each time the strain rate or target strain level was increased. During the cycles with the fast strain rate, all samples exhibited an overshoot beyond the target strain level, whereas at the slow strain rates, the peak strain matched the target strain level as per the protocol. A list of the median and IQR of the ${\tilde{E}}_{0/60}^{F}$ of all samples is shown in Table 3. For comparability with our previous study (Illi et al., 2023), we focused on those results, but a complete list of results for all strain ranges can be found in Supplementary Annex Table S1 for the slow rates and Supplementary Annex Table S2 for the fast rates.

**TABLE 3:** Median and IQR of the elastic moduli 0%–60% fast of all tested structures.

Structure	${\tilde{E}}_{0/60}^{F}$ [N/mm^2^]		
	Lower IQR	Median	Upper IQR
L-00°-00%-3-0	0.273	0.278	0.281
L-00°-00%-4-0	0.208	0.215	0.229
L-00°-00%-5-0	-	-	-
L-30°-00%-3-2	0.189	0.192	0.194
L-30°-00%-4-0	0.160	0.161	0.162
L-60°-00%-3-2	-	-	-
L-60°-00%-4-0	0.176	0.176	0.180
H-00°-20%-8-1	0.197	0.200	0.202
H-00°-40%-8-1	0.196	0.198	0.199
H-30°-20%-8-1	0.193	0.193	0.194
H-30°-40%-8-1	0.195	0.196	0.199
S-00°-17%-3-2	-	-	-
S-00°-17%-4-0	0.217	0.220	0.222
S-00°-30%-3-2	-	-	-
S-00°-30%-4-0	0.248	0.249	0.250
S-30°-17%-3-2	-	-	-
S-30°-17%-4-0	0.221	0.225	0.227
S-30°-30%-3-2	-	-	-
S-30°-30%-4-0	0.218	0.222	0.225
P-00°-00%-0-0	1.042	1.044	1.049

Lower interquartile range (IQR), median, and upper IQR of the elastic moduli for the different sample structures, measured over a strain range of 0%–60%, at fast deformation and relaxation speeds of 500 and 250 mm/min, respectively (corresponding to strain rates of ∼30 and ∼15%/s). Results of Agilus30 Clear-Along (Illi et al., 2023) reprinted as reference for pure Agilus, denoted as P-00°-00%-0-0.

### 3.1 First step—stiffness

**FIGURE 7:**
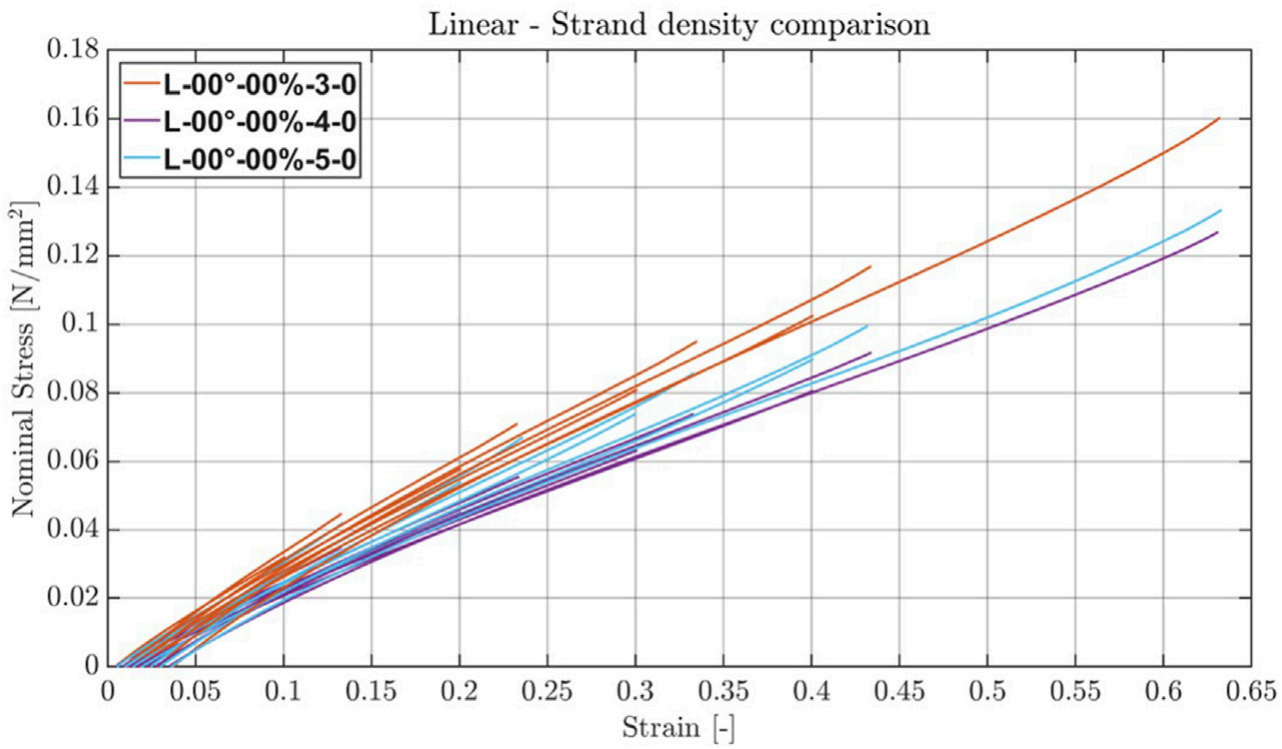
Stress–strain plots of linear structure with varying strand density. Averaged stress–strain plots of the last cycles per stage of the linear structures with 0° orientation and 0% recruitment point for three different strand densities.

The linear strand structures with orientation 0° and varying strand density showed an almost linear behavior after a strain of 10%–20%, as depicted in Figure 7. The ${\tilde{E}}_{0/60}^{F}$ value was 0.278 N/mm^2^ for the highest strand density of 3, 0.215 N/mm^2^ for the strand density of 4, and 0.232 N/mm^2^ for the strand density of 5, with sample 5 of this structure rupturing completely, leading to no usable last cycle for the last stage for this sample. Furthermore, the curves of the other samples imply that the internal structure was damaged during the first cycle of this stage.

### 3.2 Second step—anisotropy

**FIGURE 8:**
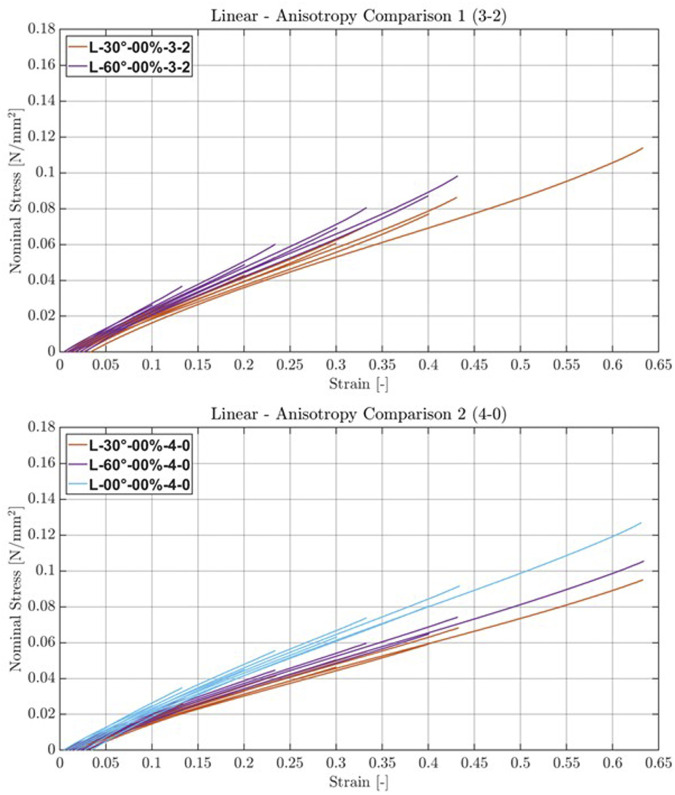
Stress–strain plots of linear structures with varying orientations. Averaged stress–strain plots of the last cycles per stage of the linear structure for two different orientations and two densities. Comparison 1: linear structure with a strand density of 3 and a layer gap of 2 voxels. Comparison 2: linear structure with a strand density of 4 and no layer gap.

The linear strand structures with a strand density of 3 and a layer gap of 2 showed an almost linear behavior after a strain of 10%, as depicted in Figure 8 (top; Comparison 1). The ${\tilde{E}}_{0/60}^{F}$ value was 0.192 N/mm^2^ for the ±30° strand orientation. For the ±60° strand orientation, ${\tilde{E}}_{0/60}^{F}$ was not calculated as all samples ruptured internally during the first cycle of that last stage. The linear strand structures with a strand density of 4 and a layer gap of 0 showed an almost linear behavior after a strain of 10%, with the above-mentioned observations, as depicted in Figure 8 (bottom; Comparison 2). The ${\tilde{E}}_{0/60}^{F}$ value was 0.161 N/mm^2^ for the ±30° strand orientation, and for ±60°, it was 0.176 N/mm^2^. None of the samples failed, and there was almost no variability between the samples. For the samples with the structure L-60°-00%-3-2 in stage 9, ${\tilde{E}}_{0/60}^{F}$ was not evaluated due to internal rupture in all samples.

### 3.3 Third step—strain hardening

#### 3.3.1 Sinusoidal organization

The sinusoidal structures with a strand density of 3 and a layer gap of 2 showed an almost linear behavior after a strain of 10%. ${\tilde{E}}_{0/60}^{F}$ was calculated neither for the 0° strand orientation nor for the ±30° strand orientations for both recruitment points of 17% and 30% strain as all samples ruptured internally during the first cycle of that last stage.

The sinusoidal structures with a strand density of 4 and a layer gap of 0 showed an almost linear behavior after a strain of 5%. In the case of a designed recruitment point at 17% strain and 0° orientation, the ${\tilde{E}}_{0/60}^{F}$ value was 0.220 N/mm^2^, with the exclusion of Sample 4, as it completely ruptured during the first cycle of the last stage. With the same recruitment point and ±30° orientation, the ${\tilde{E}}_{0/60}^{F}$ value was 0.225 N/mm^2^. For the designed fiber recruitment point at 30% strain, the ${\tilde{E}}_{0/60}^{F}$ value was 0.249 N/mm^2^ for the 0° strand orientation, and for ±30°, it was 0.222 N/mm^2^.

For all cases, no correlation between the designed fiber recruitment points and the course of the stress–strain curve was detectable.

#### 3.3.2 Helical organization

The helical structures with a double-helix diameter of 8 and a gap of 1 around the double helices showed an almost linear behavior after a strain of 10%. In the case of a designed recruitment point at 20% strain, the ${\tilde{E}}_{0/60}^{F}$ value was 0.200 N/mm^2^ for the 0° strand orientation, and for ±30°, it was 0.193 N/mm^2^. For the designed fiber recruitment point at 40% strain, the ${\tilde{E}}_{0/60}^{F}$ value was 0.198 N/mm^2^ for the 0° strand orientation and 0.196 N/mm^2^ for the ±30° orientation.

For all cases, no correlation between the designed fiber recruitment points and the course of the stress–strain curve was detectable.

**FIGURE 9:**
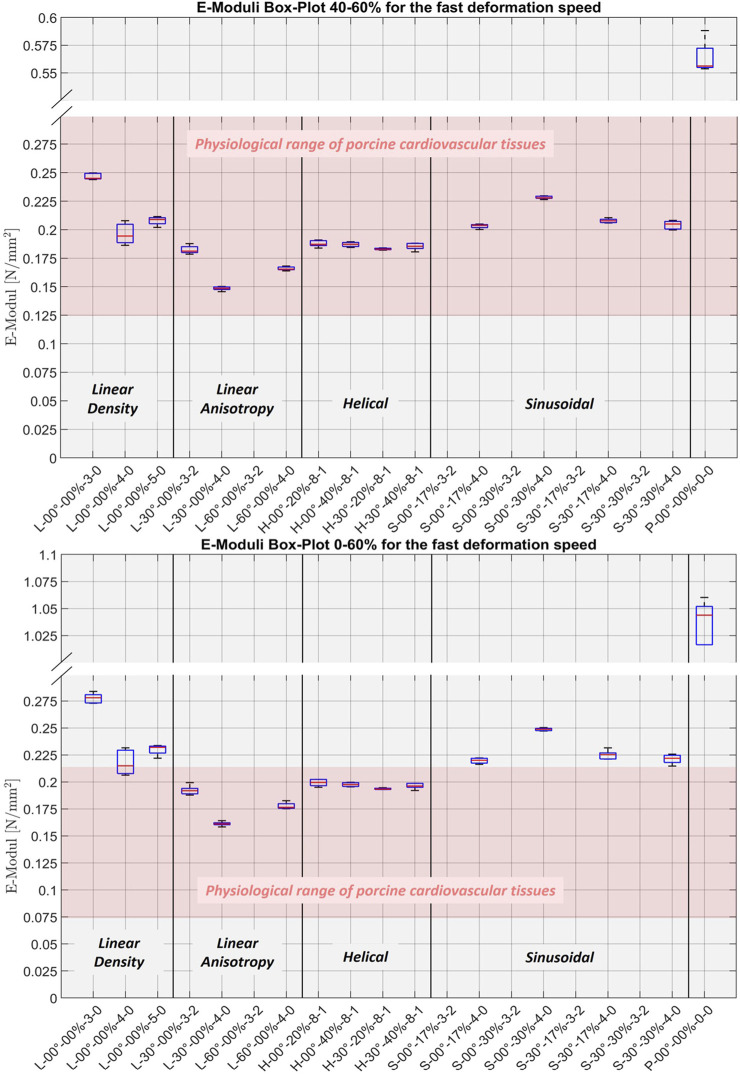
Boxplots of the elastic moduli (40%–60% and 0%–60%) for the fast deformation speed. Upper: boxplot of the elastic moduli for the strain range of 40%–60%, based on the last cycle of the last stage (10 cycles to 60%) for each sample. Lower: boxplot of the elastic moduli for the strain range of 0%–60%, based on the last cycle of the last stage (10 cycles to 60%) for each sample. The pink area marks the physiological range of porcine cardiovascular tissues for the same range. Additionally, the results of Agilus30 Clear-Along have been added as reference for pure Agilus30 Clear, denoted as P-00°-00%-0-0, both based on our previous study (Illi et al., 2023).

**FIGURE 10:**
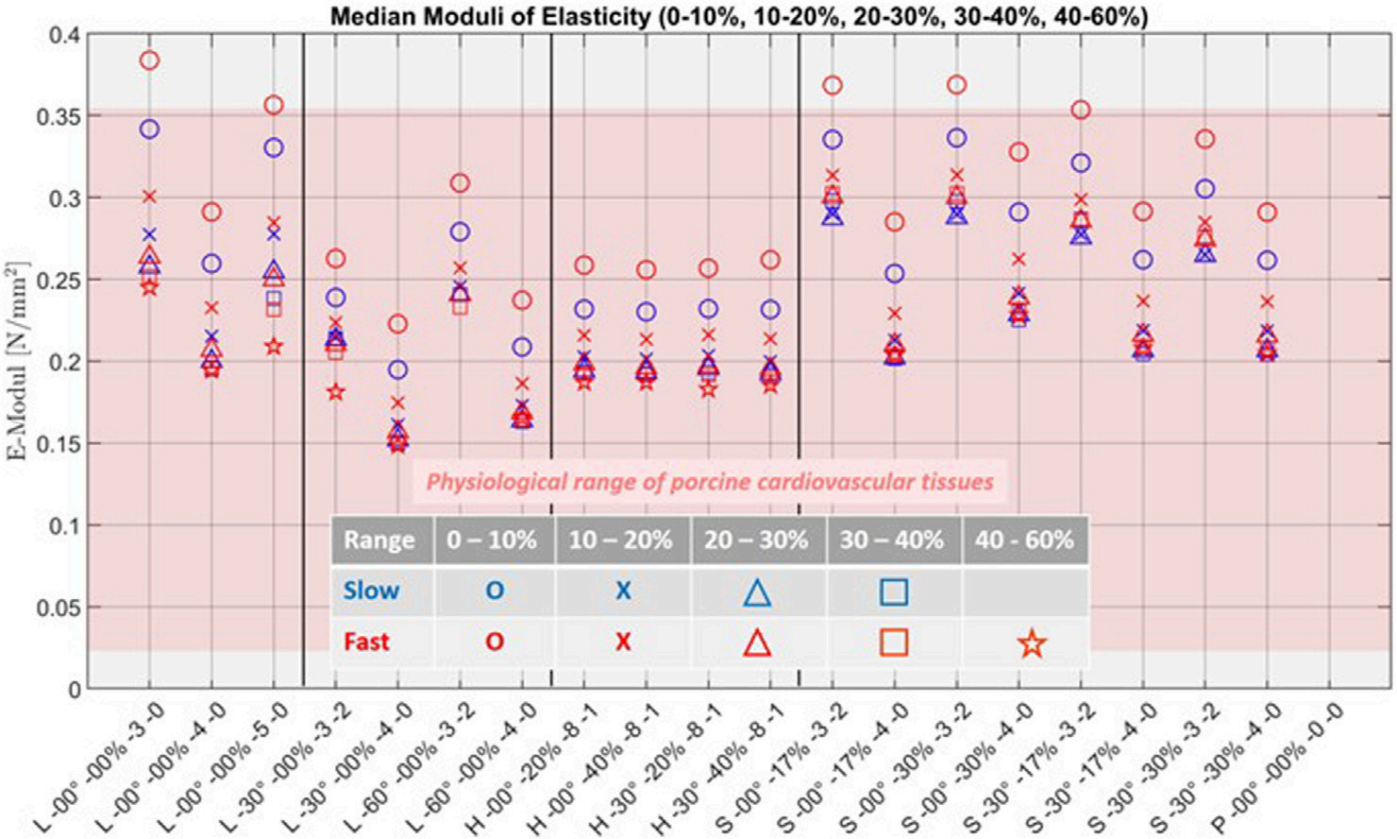
Graphical representation of the median elastic moduli and its progression for all stages and all tested samples, with the marked physiological range of porcine cardiovascular tissues highlighted in pink and the reference of pure Agilus30 Clear, P-00°-00%-0-0, corresponding to Agilus30 Clear-Along, both based on our previous study (Illi et al., 2023). The results of the pure Agilus30 Clear reference are out of scope for all ranges.

Figure 9 shows the boxplots for the 40%–60% and 0%–60% strain ranges at the fast strain rate for all structures. Figure 10 presents the plotted median elastic moduli of all structures, combined across all strain ranges and rates.

For the boxplots and the plot of the median elastic moduli, we marked the physiological range of porcine cardiovascular tissue for the corresponding strain ranges from our previous study (Illi et al., 2023). Additionally, we also incorporated the previous results of Agilus30 Clear-Along (Illi et al., 2023) into these plots as a reference for pure Agilus, labeled as P-00°-00%-0-0.

## 4 Discussion

### 4.1 Voxel-printing concept

In this study, using a stepwise approach, we were able to develop 3D voxel-printed samples with structures that more closely replicate the mechanical properties and behavior of porcine cardiovascular tissues.

#### 4.1.1 First step—stiffness

When comparing the linear structures with 0° orientation and no layer gap (0), the samples with a strand density of 3 exhibit, on average across all strain ranges for both strain rates, a 1.3-times higher modulus of elasticity than those with a strand density of 4, while the cross-sectional material density of the whole structure is less than ∼5% higher when incorporating the envelope as well. On the other hand, the samples with a strand density of 5 exhibited higher moduli of elasticity across all strain ranges and both strain rates compared to those with a strand density of 4. This is counterintuitive, but a closer inspection of the raw data revealed that all samples showed damage during the last stage, implying a lower ultimate strain. The following detailed inspection of the samples showed the same printing failures in all samples, where the strands were bundled together in blocks, with large gaps in between. This happened most likely due to a transcription error when loading the print files on the printer as the print files showed no such pattern.

#### 4.1.2 Second step—anisotropy

The samples with a strand density of 3 and a layer gap of 2 exhibited higher elastic moduli up to a strain of 40%. However, they tended to sustain damage during the first cycle when subjected to 60% strain in the last stage. Regarding orientation, both L-30°-00%-4-0 and L-30°-00%-3-2 have lower average moduli of elasticity than L-00°-00%-4-0, implicating different material behavior even though the material density is similar (L-30°-00%-3-2 vs. L-00°-00%-4-0) or identical (L-30°-00%-4-0 vs. L-00°-00%-4-0). The similar, and in some cases slightly higher, elastic moduli of the ±60° structures compared to those of the ±30° structures is counterintuitive, especially as further inspection showed no printing inconsistencies. One possible explanation lies in the narrow width of the sample; with a ±60° strand orientation, most strands connect the walls of the envelope, thereby reinforcing it. To mitigate this effect and better explore the anisotropic potential, measurements should be conducted with either an open envelope on the sides or no envelope at all. A biaxial testing setup would be required for a more thorough investigation.

#### 4.1.3 Third step—strain hardening

##### 4.1.3.1 Sinusoidal

Most samples with a strand density of 3 and a layer gap of 2 (3-2) experienced internal ruptured or complete failure during the last stage (0%–60% fast). Thus, no modulus of elasticity was calculated for the 40%–60% or 0%–60% strain ranges for any of those four sinusoidal structures with that strand configuration (3-2). Nevertheless, for the remaining strain ranges, we measured the lower moduli of elasticity for the two ±30° orientations when comparing them with their equivalent 0° structures. The designed recruitment point appeared to have no influence on the stress–strain behavior, implying either no effect or a significantly small effect relative to the general stress level.

The four sinusoidal structures with a strand density of 4 and no layer gap (0) had no internal ruptures, and only one sample failed during the last stage (0%–60% fast). This supports the initial statement in Section 4.1 that the cross-linking, due to the lack of a layer gap, increases the ultimate strain of the structures. However, also for those structures, we observed no influence of the designed recruitment point on the course of the stress–strain curve.

##### 4.1.3.2 Helical

For the four helical structures, neither the orientation (0° vs. ±30°) nor the designed recruitment point showed any influence, resulting in similar, almost identical results. Nevertheless, these structures exhibited an almost completely linear behavior, with no indications of internal rupture and a very low stress response.

The lack of any effect of the designed recruitment point on the sinusoidal and helical organizations is counterintuitive since the accurate printing of the structures was confirmed (see Figure 5D). The concept of helices and sinusoidal structures having a non-linear response under elongation corresponds to basic mechanics and has been proven in a similar application, with the same printing technology but on a larger structure scale, without voxel-printing and with different materials (Wang et al., 2016). It is most likely that before the designed recruitment point, the stress response is completely dominated by the envelope. By the time the strands are expected to contribute, the strain in the envelope may already be so high that the influence of the strands is not detectable.

In general, most of the internal ruptures and complete sample failures occurred on samples with a strand density of 3 and a layer gap of 2. This implies that the cross-linking between the strands within the samples with a strand density of 4 and no layer gap (0) helps in the distribution of the load. Furthermore, when comparing the same result for two structures that differ only in strand density and layer gap (same strain rate, target strain level, evaluation range, strand orientation, and recruitment point), the structures with a strand density of 4 and no layer gap (0) always have lower elastic moduli (see Figure 10). This is the case, despite the similar cross-sectional material density of both strand arrangements (4-0 and 3-2), which are very comparable at 36.5 mm^2^ for 4-0 and 37.4 mm^2^ for 3-2 structures. Moreover, certain samples tended to fail progressively due to internal ruptures that, in some cases, persisted until the end of the loading protocol, outer envelope remained intact.

### 4.2 Comparison with other phantom materials

Compared to our previous results of cardiovascular phantom materials (Illi et al., 2023), we have a significant reduction in elastic moduli relative to commonly used printing materials and silicones. Additionally, the stress–strain response is more linear and exhibits reduced strain softening compared to that of pure Agilus30 Clear. Moreover, the anisotropic behavior across all structures, resembling that of soft tissues, represents a previously unreproducible or cost-intensive feature in conventional phantom materials. The range of elasticity and anisotropic behavior can be controlled locally, a capability that is either highly limited or entirely absent in conventional materials. This implies that the voxel-printing approach and the tested structures may serve as a viable alternative for applications requiring a softer, more compliant material response than Agilus30 Clear, combined with anisotropic behavior and locally tunable properties, such as artificial tissue materials in cardiovascular phantom applications.

An exemplary translation of this technology into a patient-specific phantom would be an aortic root model, including the coronary arteries. Voxel printing would allow for a more realistic mechanical representation of the aortic wall of the phantom by mimicking the compliance of the tissue with the lower modulus of elasticity and strand orientation according to the dominant orientation in the tissue. This would allow separate adjustments of the material for the aorta and the coronary arteries and for different pathologies or stages of diseases. This could enable a more accurate representation of *in vivo* conditions, for example, in hemodynamic testing, where the aorta would dilate more realistically (Illi et al., 2024). It would also improve the planning, testing, and investigation of transaortic valve implantations by more accurately reproducing the interaction between the expanding stent and the aortic wall than currently used materials. The same assigning of matching structures could be performed in coronary arteries. This would aid in the investigation of stenosis severity (Illi et al., 2024), plaque assessment, and the simulation of effective balloon angioplasty interventions. Moreover, the process remains compatible with the integration of stiffer PolyJet materials, like VeroPureWhite, enabling the representation of calcified tissue regions, consistent with current practices (Illi et al., 2022; Bernhard et al., 2022).

Regardless of the application, thin (0.2–0.4 mm) closed inner and outer layers will always be needed, either as a water barrier to distribute the load of an implant or tool onto the structure or to ensure the integrity of the structure within them. With respect to thinner vessels, the buildup and structure of the voxel-printing process might need to be adapted to the spatial limitations. In this case, a multi-layer helical structure with strands wrapping around the lumen of the vessels and layer-by-layer changing orientation might offer a solution.

Finally, other materials and techniques also show promise, such as polyethylene glycol hydrogels and electrospun polycaprolactone. These materials are primarily used in tissue engineering, and some are even compatible with 3D printing. However, they are less suitable for phantom applications as they do not readily allow the direct fabrication of complex geometries and creating local variations in mechanical properties is challenging or unfeasible. Additionally, their production often involves cell incubation for scaffold integration and requires specialized handling due to their bioactive nature and the need to maintain cell viability.

### 4.3 Comparison with porcine cardiovascular tissues

The presented voxel-printing structures can be used to create softer, more realistic phantom materials, which can even mimic different fiber orientations and, thus, the anisotropy of soft tissues. Nevertheless, to further improve the realistic representation of soft tissues, the non-linear strain hardening behavior due to fiber recruitment is crucial. Wang et al. (2016) investigated a similar PolyJet metamaterial approach with similar organizations to mimic the strain-hardening behavior of soft tissues. However, without our voxel-printing concept, at a larger structure scale and with stiffer materials, they were able to achieve the desired behavior in their targeted strain range (0%–8%), although with five- to ten-times higher moduli of elasticity.

### 4.4 Outlook

To investigate the lack of influence of the designed recruitment point, further tests should be performed to reduce the impact of the sample geometry (envelope)— for example, with open envelope sides, perforated envelope sides, and outer face or no envelope at all. This would also address the issue of internal rupture in the sample structures and the subsequent detachment from the envelope. Furthermore, the width of the sample should also be increased (plane-strain tests) to better represent the intended application. Although this may reduce the quality of the comparison to previous results, it is necessary to further characterize the material.

Additionally, a parameter sensitivity study should be conducted to verify whether the appropriate parameters and values were selected before proceeding with testing.

Furthermore, investigating local strain measurements—for example, using digital image correlation—on the surface of the envelope or within the internal stranded structures, can reveal subtle effects and localized strain behaviors that are not captured in bulk stress–strain curves.

To more accurately mimic cardiovascular soft tissues while not having to perform hundreds of sample tests to further optimize the parameters, these data should be used to build and validate a numerical simulation to calculate the specific parameters for a given application and tissue.

Such a computational model could also help investigate the strand recruitment behavior and analyze failure modes and points of failure.

For the final comparison to cardiovascular tissue, a multiaxial testing study of the target tissues and their corresponding voxel-printing structures should be performed with the above-mentioned multiaxial and local strain measurement.

Additionally, to increase the usability and allow testing of the presented concept in full phantom applications, the design and modeling process should be streamlined to minimize the complexity of the process and optimize computational resources. The entire process, from anatomical model import and structural calculation to generation and direct code export for the printer, should be ideally integrated into a single software platform. This approach would help avoid intermediate conversions and would allow a fully consistent voxel-to-voxel workflow (Illi et al., 2022). In applications requiring phantom reuse or long-term assessments such as endurance or fatigue testing, it is essential to evaluate the durability and aging characteristics of both the materials and the structural design.

Finally, the approach should be validated in a simple application, where tissues and artificial voxel-printing materials can be tested as closely as possible, e.g., in dynamic dilatation tests of vessels and tubes.

### 4.5 Limitations

Even though the sample geometry was optimized to reduce the influence of the outer Agilus30 shell, further reduction is not possible as the shell is needed during the removal of the support material to protect the voxel-printing structure from damage. Chemically dissolving the support could alter the mechanical behavior. Furthermore, one important application of cardiovascular phantoms with physiological compliance is for hemodynamic testing, where the water-tightness property is required.

Moreover, the mechanical properties of cardiovascular tissues are much more complex than the scope of the performed test. Although our protocol aims to simulate different conditions, it inevitably falls short of capturing the entire mechanical response spectrum of living tissues. No artificial, non-tissue-engineered material can fully replicate the behavior of cardiovascular tissues under all possible states and conditions. Thus, we believe that our chosen parameters provide a reasonable approximation of physiological conditions for an assessment.

An indirect limitation to our study is the reliance on deceased porcine cardiovascular tissue as a reference (Illi et al., 2023), rather than living human tissue (Martin et al., 2011). The differences can influence the mechanical response and, therefore, the applicability of our findings to human cardiovascular tissues. Nonetheless, we can infer that the differences between porcine and human tissues are smaller than the observed disparities between the current artificial phantom materials and porcine tissue (Ferrara et al., 2016). With the materials now presented, further investigation into this aspect is feasible and should be performed.

To properly investigate and accurately measure anisotropic material behavior, a biaxial tension testing setup and biaxial strain measurement system would be needed. This way the anisotropic behavior can be properly investigated, accounting for local behavior, sample narrowing, and compound effects. Our approach of comparing results from samples with different orientations, due to limited equipment, is only a minimal starting point. Nevertheless, our results show a clear anisotropic behavior compared to the isotropic behavior of Agilus30 Clear, independent of printing orientation in our previous investigation (Illi et al., 2023).

In our study, sample deformation corresponded to the vice grip displacement of the testing setup. This displacement can differ from actual sample deformation; this error is negligible as the setup is designed for 100-times higher forces. Since our focus is on cardiovascular phantoms, the materials used in this study do not need to be biocompatible. This allows us to use these materials and the corresponding multi-material voxel-printing process despite their lack of biocompatibility.

## 5 Conclusion

The novel 3D voxel-printing material approach resulted in reduced elastic modulus, anisotropic behavior, and strain hardening, providing a much closer representation of the mechanical behavior of porcine cardiovascular tissues compared to other available phantom materials. However, there is still significant potential for optimization through further exploration of mimicking fiber recruitment.

## Data Availability

The raw data supporting the conclusions of this article will be made available by the authors, without undue reservation.

## Abbreviations

AM, Additive manufacturing; 3DPSP, 3-dimensional patient-specific phantom; ${\tilde{E}}_{0/60}^{F}$ , median elastic modulus 0%–60% fast.

## References

[B1] Bernhard B. Illi J. Gloeckler M. Pilgrim T. Praz F. Windecker S. (2022). Imaging-based, patient-specific three-dimensional printing to plan, train, and guide cardiovascular interventions: a systematic review and meta-analysis. Heart Lung Circ. 31 (9), 1203–1218. 10.1016/j.hlc.2022.04.052 35680498

[B2] Dokos S. Smaill B. H. Young A. A. LeGrice I. J. (2002). Shear properties of passive ventricular myocardium. Am. J. Physiol. Heart Circ. Physiol. 283 (6), H2650–H2659. 10.1152/ajpheart.00111.2002 12427603

[B3] El Sabbagh A. Eleid M. F. Matsumoto J. M. Anavekar N. S. Al‐Hijji M. A. Said S. M. (2018). Three-dimensional prototyping for procedural simulation of transcatheter mitral valve replacement in patients with mitral annular calcification. Catheter Cardiovasc Interv. 92 (7), E537–E549. 10.1002/ccd.27488 29359388

[B4] Emig R. Zgierski-Johnston C. M. Timmermann V. Taberner A. J. Nash M. P. Kohl P. (2021). Passive myocardial mechanical properties: meaning, measurement, models. Biophys. Rev. 13 (5), 587–610. 10.1007/s12551-021-00838-1 34765043 PMC8555034

[B5] Ferrara A. Morganti S. Totaro P. Mazzola A. Auricchio F. (2016). Human dilated ascending aorta: mechanical characterization via uniaxial tensile tests. J. Mech. Behav. Biomed. Mater. 53, 257–271. 10.1016/j.jmbbm.2015.08.021 26356765

[B6] Flamini V. Kerskens C. Simms C. Lally C. (2013). Fibre orientation of fresh and frozen porcine aorta determined non-invasively using diffusion tensor imaging. Med. Eng. Phys. 35 (6), 765–776. 10.1016/j.medengphy.2012.08.008 22998893

[B7] Illi J. Bernhard B. Nguyen C. Pilgrim T. Praz F. Gloeckler M. (2022). Translating imaging into 3D printed cardiovascular phantoms: a systematic review of applications, technologies, and validation. JACC Basic Transl. Sci. 7 (10), 1050–1062. 10.1016/j.jacbts.2022.01.002 36337920 PMC9626905

[B8] Illi J. Ilic M. Stark A. W. Amstutz C. Burger J. Zysset P. (2023). Mechanical testing and comparison of porcine tissue, silicones and 3D-printed materials for cardiovascular phantoms. Front. Bioeng. Biotechnol. 11, 1274673. 10.3389/fbioe.2023.1274673 38107617 PMC10725245

[B9] Illi J. Stark A. W. Ilic M. Soares Loureiro D. Obrist D. Shiri I. (2024). Hemodynamic relevance evaluation of coronary artery anomaly during stress using FFR/IVUS in an artificial twin. JACC Case Rep. 30, 102729. 10.1016/j.jaccas.2024.102729 39822811 PMC11733583

[B10] Kim J. H. Park C. K. Park J. E. Lee J. M. (2021). 3D print material study to reproduce the function of pig heart tissue. Technol Health Care. 29 (S1), 27–34. 10.3233/THC-218003 33682742 PMC8150471

[B11] Li Z. Luo T. Wang S. Jia H. Gong Q. Liu X. (2022). Mechanical and histological characteristics of aortic dissection tissues. Acta Biomater. 146, 284–294. 10.1016/j.actbio.2022.03.042 35367380

[B12] Lokshin O. Lanir Y. (2009). Viscoelasticity and preconditioning of rat skin under uniaxial stretch: microstructural constitutive characterization. J. Biomech. Eng. 131 (3), 031009. 10.1115/1.3049479 19154068

[B13] Martin C. Pham T. Sun W. (2011). Significant differences in the material properties between aged human and porcine aortic tissues. Eur J Cardiothorac Surg. 40 (1), 28–34. 10.1016/j.ejcts.2010.08.056 21177118 PMC3080441

[B14] Nemavhola F. (2021). Study of biaxial mechanical properties of the passive pig heart: material characterisation and categorisation of regional differences. Int. J. Mech. Mater. Eng. 16 (1), 6. 10.1186/s40712-021-00128-4

[B15] Park C-K. Kim J. (2022). Development of a three-dimensional-printed heart model replicating the elasticity, tear resistance, and hardness of pig heart using agilus and tango. J. Mech. Med. Biol. 22 (03), 2240007. 10.1142/s0219519422400073

[B16] Peña E. Peña J. A. Doblaré M. (2009). On the Mullins effect and hysteresis of fibered biological materials: a comparison between continuous and discontinuous damage models. Int. J. Solids Struct. 46 (7), 1727–1735. 10.1016/j.ijsolstr.2008.12.015

[B17] Scanlan A. B. Nguyen A. V. Ilina A. Lasso A. Cripe L. Jegatheeswaran A. (2018). Comparison of 3D echocardiogram-derived 3D printed valve models to molded models for simulated repair of pediatric atrioventricular valves. Pediatr. Cardiol. 39 (3), 538–547. 10.1007/s00246-017-1785-4 29181795 PMC5831483

[B18] Schriefl A. J. Reinisch A. J. Sankaran S. Pierce D. M. Holzapfel G. A. (2012). Quantitative assessment of collagen fibre orientations from two-dimensional images of soft biological tissues. J. R. Soc. Interface 9 (76), 3081–3093. 10.1098/rsif.2012.0339 22764133 PMC3479916

[B19] Sommer G. Schriefl A. J. Andrä M. Sacherer M. Viertler C. Wolinski H. (2015). Biomechanical properties and microstructure of human ventricular myocardium. Acta Biomater. 24, 172–192. 10.1016/j.actbio.2015.06.031 26141152

[B20] Voigt J. U. Cvijic M. (2019). 2- and 3-dimensional myocardial strain in cardiac health and disease. JACC Cardiovasc Imaging 12 (9), 1849–1863. 10.1016/j.jcmg.2019.01.044 31488253

[B21] Wang K. Wu C. Qian Z. Zhang C. Wang B. Vannan M. A. (2016). Dual-material 3D printed metamaterials with tunable mechanical properties for patient-specific tissue-mimicking phantoms. Addit. Manuf. 12, 31–37. 10.1016/j.addma.2016.06.006

[B22] Yoo S. J. Spray T. Austin E. H. 3rd Yun T. J. van Arsdell G. S. (2017). Hands-on surgical training of congenital heart surgery using 3-dimensional print models. J. Thorac. Cardiovasc. Surg. 153 (6), 1530–1540. 10.1016/j.jtcvs.2016.12.054 28268011

[B23] Zhalmuratova D. La T. G. Yu K. T. T. Szojka A. R. A. Andrews S. H. J. Adesida A. B. (2019). Mimicking “J-Shaped” and anisotropic stress-strain behavior of human and porcine aorta by fabric-reinforced elastomer composites. ACS Appl. Mater. Interfaces 11 (36), 33323–33335. 10.1021/acsami.9b10524 31464413

