# Supplementary material for: Mechanical properties of 3D voxel-printed materials for cardiovascular tissue imitation
Source: Front Bioeng Biotechnol. 2025 May 30;13:1569553. doi: 10.3389/fbioe.2025.1569553 (PMC12162977; doi:10.3389/fbioe.2025.1569553)
Supplement: Supplementary file 1 [file Supplementaryfile1.docx]

**Annex**

**I Material and Methods**

**I.a Sample design**

**Strand density and layer gap**

Due to the used calculations for the voxel structures, the strand density value is inverse proportional to the material density. The higher the strand density value the fewer strands are in the structure.

To investigate the influence of cross-linking between the strands, we selected the parameter pairs to maintain a comparable cross-sectional material density. Leading to X-XX°-XX%-4-0 structures with a strand density 4 and a layer gap of 0 voxels and the X-XX°-XX%-3-2 structures with a strand density 3 and a layer gap of 2 voxels. Due to the discrete voxel domain, this leads to ratios 0.25 for 4-0 and 0.266 for 3-2 without the envelope. With the envelope this corresponds to cross-sectional material areas of 36.5 mm^2^ for 4-0 and 37.4 mm^2^ for 3-2, whilst to total cross-sectional area of the samples was 100 mm^2^.

**Strand orientation**

For the fibre orientation in vessels we found literature values of ±12-33° [22] to ±36-38° [23], depending on the tested cardiovascular tissue, location, setup and sample preparation. Thus, we decided to use ±30°, corresponding with the loading axis in circumferential direction of a vessel. Furthermore, we also used ±60°, which would correspond with the loading axis in longitudinal axis of a vessel, and which allows us to compare two orthogonal orientations for the anisotropy.

**Sinusoidal organisation**

The strain value for the recruitment point of the sinusoidal organisation depends on the amplitude and the wavelength of the strands. In the voxel domain these values are discrete and can only be multiples of printing voxel, or in our case of binning elements. For the tested structures we fixed the amplitude to 4 binning elements and selected the wavelength accordingly, with 28 elements (~8 cycles over the sample length) for a recruitment point of ~17% and 21 elements (~11 cycles over the sample length) for a recruitment point of ~30%.

**Helical organisation**

For the helical organisation, the recruitment point is depending on the diameter of the helix and the pitch. The diameter has a direct lower limit, due to the strand thickness, of 1.6 binning elements, to avoid intersections between the helices and an indirect upper limit of 1.8 to 2 binning elements, to keeping the strand density above 5. The strand density then also required the helix gap (separation between the double helices) to be as small as possible, whilst avoid any cross-linking. With these limitations and the requirement to have a fibre recruitment point in the range from 0 to 60% strain, we had to deviate for this structure from the binning voxels as smallest discrete volume unit and to use native printing voxels instead.

This led to a diameter of 1.8 binning elements or 9 printing voxels for all helical structures and a helix gap of 0.1 binning elements or 1 native printing voxel. The pitch was then used to create the fibre recruitment points at ~20% strain with 9 and at ~40% with 6 printing voxels.

The ensure the fidelity of the samples to the designed structures, one sample of each helical structure was dissected, dyed and analysed under a microscope (Figure 5 (D)).

**I.b Mechanical testing**

For a better understanding of the used testing protocol, Table 2 lists all cycles with the used parameters, stage, deformation speed / strain rate, target strain level and evaluated cycle. Figure 6 shows the time-strain plot, that results from this protocol.

**Annex Tables**

|  |  | Elastic moduli slow [N/mm^2^] | | |
| --- | --- | --- | --- | --- |
| Structure | Strain [%] | Lower IQR | Median | Upper IQR |
| L-00°-00%-3-0 | 0-10 | 0.335 | 0.342 | 0.345 |
|  | 10-20 | 0.277 | 0.277 | 0.282 |
|  | 20-30 | 0.256 | 0.258 | 0.262 |
|  | 30-40 | 0.249 | 0.252 | 0.255 |
| L-00°-00%-4-0 | 0-10 | 0.249 | 0.260 | 0.281 |
|  | 10-20 | 0.206 | 0.215 | 0.229 |
|  | 20-30 | 0.192 | 0.200 | 0.211 |
|  | 30-40 | 0.188 | 0.195 | 0.206 |
| L-00°-00%-5-0 | 0-10 | 0.309 | 0.330 | 0.345 |
|  | 10-20 | 0.261 | 0.278 | 0.290 |
|  | 20-30 | 0.246 | 0.255 | 0.263 |
|  | 30-40 | 0.233 | 0.238 | 0.245 |
| L-30°-00%-3-2 | 0-10 | 0.237 | 0.239 | 0.244 |
|  | 10-20 | 0.214 | 0.215 | 0.220 |
|  | 20-30 | 0.209 | 0.213 | 0.216 |
|  | 30-40 | 0.209 | 0.214 | 0.215 |
| L-30°-00%-4-0 | 0-10 | 0.190 | 0.195 | 0.195 |
|  | 10-20 | 0.158 | 0.161 | 0.162 |
|  | 20-30 | 0.149 | 0.152 | 0.152 |
|  | 30-40 | 0.148 | 0.150 | 0.150 |
| L-60°-00%-3-2 | 0-10 | 0.273 | 0.279 | 0.283 |
|  | 10-20 | 0.243 | 0.245 | 0.251 |
|  | 20-30 | 0.239 | 0.241 | 0.245 |
|  | 30-40 | 0.238 | 0.241 | 0.243 |
| L-60°-00%-4-0 | 0-10 | 0.208 | 0.209 | 0.209 |
|  | 10-20 | 0.170 | 0.173 | 0.174 |
|  | 20-30 | 0.159 | 0.164 | 0.164 |
|  | 30-40 | 0.158 | 0.163 | 0.164 |
| H-00°-20%-8-1 | 0-10 | 0.228 | 0.232 | 0.243 |
|  | 10-20 | 0.201 | 0.203 | 0.210 |
|  | 20-30 | 0.193 | 0.194 | 0.202 |
|  | 30-40 | 0.191 | 0.193 | 0.200 |
| H-00°-40%-8-1 | 0-10 | 0.226 | 0.230 | 0.239 |
|  | 10-20 | 0.197 | 0.201 | 0.207 |
|  | 20-30 | 0.190 | 0.193 | 0.199 |
|  | 30-40 | 0.189 | 0.192 | 0.196 |
| H-30°-20%-8-1 | 0-10 | 0.228 | 0.232 | 0.234 |
|  | 10-20 | 0.199 | 0.203 | 0.204 |
|  | 20-30 | 0.191 | 0.195 | 0.196 |
|  | 30-40 | 0.190 | 0.193 | 0.194 |
| H-30°-40%-8-1 | 0-10 | 0.227 | 0.232 | 0.238 |
|  | 10-20 | 0.198 | 0.200 | 0.207 |
|  | 20-30 | 0.190 | 0.193 | 0.198 |
|  | 30-40 | 0.189 | 0.191 | 0.196 |
| S-00°-17%-3-2 | 0-10 | 0.325 | 0.335 | 0.337 |
|  | 10-20 | 0.282 | 0.290 | 0.291 |
|  | 20-30 | 0.282 | 0.287 | 0.288 |
|  | 30-40 | 0.294 | 0.298 | 0.299 |
| S-00°-17%-4-0 | 0-10 | 0.253 | 0.254 | 0.258 |
|  | 10-20 | 0.210 | 0.213 | 0.216 |
|  | 20-30 | 0.197 | 0.202 | 0.204 |
|  | 30-40 | 0.195 | 0.202 | 0.202 |
| S-00°-30%-3-2 | 0-10 | 0.330 | 0.336 | 0.337 |
|  | 10-20 | 0.287 | 0.290 | 0.292 |
|  | 20-30 | 0.285 | 0.288 | 0.288 |
|  | 30-40 | 0.296 | 0.298 | 0.299 |
| S-00°-30%-4-0 | 0-10 | 0.290 | 0.291 | 0.294 |
|  | 10-20 | 0.241 | 0.241 | 0.243 |
|  | 20-30 | 0.227 | 0.228 | 0.229 |
|  | 30-40 | 0.225 | 0.225 | 0.226 |
| S-30°-17%-3-2 | 0-10 | 0.317 | 0.321 | 0.324 |
|  | 10-20 | 0.275 | 0.277 | 0.281 |
|  | 20-30 | 0.274 | 0.275 | 0.281 |
|  | 30-40 | 0.284 | 0.286 | 0.292 |
| S-30°-17%-4-0 | 0-10 | 0.258 | 0.262 | 0.268 |
|  | 10-20 | 0.216 | 0.219 | 0.223 |
|  | 20-30 | 0.205 | 0.206 | 0.210 |
|  | 30-40 | 0.204 | 0.205 | 0.207 |
| S-30°-30%-3-2 | 0-10 | 0.296 | 0.305 | 0.314 |
|  | 10-20 | 0.259 | 0.265 | 0.270 |
|  | 20-30 | 0.259 | 0.265 | 0.267 |
|  | 30-40 | 0.271 | 0.276 | 0.278 |
| S-30°-30%-4-0 | 0-10 | 0.254 | 0.262 | 0.265 |
|  | 10-20 | 0.213 | 0.218 | 0.221 |
|  | 20-30 | 0.202 | 0.206 | 0.208 |
|  | 30-40 | 0.201 | 0.204 | 0.205 |
| P-00°-00%-0-0 | 0-10 | - | - | - |
|  | 10-20 | - | - | - |
|  | 20-30 | - | - | - |
|  | 30-40 | - | - | - |

**Annex Table 1 Median and IQR of the elastic moduli of all ranges and tested structures for the slow deformation speed**

Lower inter quartile range (IQR), Median and upper IQR of the elastic moduli for the different sample structures for the strain ranges 0-10%, 10-20%, 20-30% and 30-40% for the slow deformation and relaxation speed of 50 mm/min, respectively a strain rate of ~3 %/s. As the Agilus30Clear-Along have only be tested with the old protocol (fast), there is no data for the slow strain rate for the pure Agilus samples.

|  |  | Elastic moduli fast [N/mm^2^] | | |
| --- | --- | --- | --- | --- |
| Structure | Strain [%] | Lower IQR | Median | Upper IQR |
| L-00°-00%-3-0 | 0-10 | 0.373 | 0.384 | 0.387 |
|  | 10-20 | 0.296 | 0.301 | 0.303 |
|  | 20-30 | 0.263 | 0.264 | 0.269 |
|  | 30-40 | 0.250 | 0.252 | 0.256 |
|  | 40-60 | 0.245 | 0.245 | 0.249 |
|  | 0-60 | 0.273 | 0.278 | 0.281 |
| L-00°-00%-4-0 | 0-10 | 0.282 | 0.291 | 0.316 |
|  | 10-20 | 0.223 | 0.233 | 0.248 |
|  | 20-30 | 0.198 | 0.207 | 0.219 |
|  | 30-40 | 0.190 | 0.196 | 0.208 |
|  | 40-60 | 0.189 | 0.194 | 0.205 |
|  | 0-60 | 0.208 | 0.215 | 0.229 |
| L-00°-00%-5-0 | 0-10 | 0.339 | 0.356 | 0.369 |
|  | 10-20 | 0.275 | 0.285 | 0.293 |
|  | 20-30 | 0.244 | 0.250 | 0.256 |
|  | 30-40 | 0.227 | 0.232 | 0.236 |
|  | 40-60 | - | - | - |
|  | 0-60 | - | - | - |
| L-30°-00%-3-2 | 0-10 | 0.261 | 0.263 | 0.269 |
|  | 10-20 | 0.221 | 0.223 | 0.228 |
|  | 20-30 | 0.208 | 0.210 | 0.214 |
|  | 30-40 | 0.202 | 0.205 | 0.207 |
|  | 40-60 | 0.180 | 0.181 | 0.185 |
|  | 0-60 | 0.189 | 0.192 | 0.194 |
| L-30°-00%-4-0 | 0-10 | 0.218 | 0.223 | 0.224 |
|  | 10-20 | 0.171 | 0.175 | 0.175 |
|  | 20-30 | 0.154 | 0.157 | 0.157 |
|  | 30-40 | 0.148 | 0.151 | 0.151 |
|  | 40-60 | 0.148 | 0.149 | 0.150 |
|  | 0-60 | 0.160 | 0.161 | 0.162 |
| L-60°-00%-3-2 | 0-10 | 0.300 | 0.309 | 0.312 |
|  | 10-20 | 0.254 | 0.257 | 0.261 |
|  | 20-30 | 0.238 | 0.241 | 0.245 |
|  | 30-40 | 0.230 | 0.233 | 0.235 |
|  | 40-60 | - | - | - |
|  | 0-60 | - | - | - |
| L-60°-00%-4-0 | 0-10 | 0.235 | 0.237 | 0.240 |
|  | 10-20 | 0.186 | 0.186 | 0.188 |
|  | 20-30 | 0.167 | 0.168 | 0.170 |
|  | 30-40 | 0.161 | 0.164 | 0.165 |
|  | 40-60 | 0.165 | 0.165 | 0.167 |
|  | 0-60 | 0.176 | 0.176 | 0.180 |
| H-00°-20%-8-1 | 0-10 | 0.255 | 0.259 | 0.271 |
|  | 10-20 | 0.214 | 0.216 | 0.226 |
|  | 20-30 | 0.196 | 0.198 | 0.207 |
|  | 30-40 | 0.191 | 0.192 | 0.200 |
|  | 40-60 | 0.186 | 0.187 | 0.190 |
|  | 0-60 | 0.197 | 0.200 | 0.202 |
| H-00°-40%-8-1 | 0-10 | 0.250 | 0.256 | 0.264 |
|  | 10-20 | 0.210 | 0.213 | 0.221 |
|  | 20-30 | 0.193 | 0.196 | 0.202 |
|  | 30-40 | 0.189 | 0.192 | 0.197 |
|  | 40-60 | 0.185 | 0.187 | 0.189 |
|  | 0-60 | 0.196 | 0.198 | 0.199 |
| H-30°-20%-8-1 | 0-10 | 0.255 | 0.257 | 0.258 |
|  | 10-20 | 0.212 | 0.216 | 0.217 |
|  | 20-30 | 0.193 | 0.197 | 0.199 |
|  | 30-40 | 0.188 | 0.192 | 0.192 |
|  | 40-60 | 0.182 | 0.183 | 0.184 |
|  | 0-60 | 0.193 | 0.193 | 0.194 |
| H-30°-40%-8-1 | 0-10 | 0.254 | 0.262 | 0.266 |
|  | 10-20 | 0.212 | 0.214 | 0.220 |
|  | 20-30 | 0.194 | 0.195 | 0.201 |
|  | 30-40 | 0.188 | 0.190 | 0.195 |
|  | 40-60 | 0.184 | 0.185 | 0.188 |
|  | 0-60 | 0.195 | 0.196 | 0.199 |
| S-00°-17%-3-2 | 0-10 | 0.359 | 0.368 | 0.370 |
|  | 10-20 | 0.304 | 0.313 | 0.314 |
|  | 20-30 | 0.292 | 0.300 | 0.301 |
|  | 30-40 | 0.295 | 0.303 | 0.303 |
|  | 40-60 | - | - | - |
|  | 0-60 | - | - | - |
| S-00°-17%-4-0 | 0-10 | 0.281 | 0.285 | 0.287 |
|  | 10-20 | 0.228 | 0.229 | 0.234 |
|  | 20-30 | 0.206 | 0.210 | 0.213 |
|  | 30-40 | 0.199 | 0.204 | 0.206 |
|  | 40-60 | 0.202 | 0.204 | 0.204 |
|  | 0-60 | 0.217 | 0.220 | 0.222 |
| S-00°-30%-3-2 | 0-10 | 0.363 | 0.369 | 0.372 |
|  | 10-20 | 0.309 | 0.314 | 0.315 |
|  | 20-30 | 0.297 | 0.300 | 0.301 |
|  | 30-40 | 0.299 | 0.302 | 0.304 |
|  | 40-60 | - | - | - |
|  | 0-60 | - | - | - |
| S-00°-30%-4-0 | 0-10 | 0.325 | 0.328 | 0.330 |
|  | 10-20 | 0.262 | 0.262 | 0.264 |
|  | 20-30 | 0.238 | 0.239 | 0.240 |
|  | 30-40 | 0.229 | 0.230 | 0.231 |
|  | 40-60 | 0.228 | 0.228 | 0.229 |
|  | 0-60 | 0.248 | 0.249 | 0.250 |
| S-30°-17%-3-2 | 0-10 | 0.346 | 0.354 | 0.357 |
|  | 10-20 | 0.295 | 0.299 | 0.303 |
|  | 20-30 | 0.283 | 0.285 | 0.290 |
|  | 30-40 | 0.283 | 0.287 | 0.290 |
|  | 40-60 | - | - | - |
|  | 0-60 | - | - | - |
| S-30°-17%-4-0 | 0-10 | 0.289 | 0.291 | 0.298 |
|  | 10-20 | 0.233 | 0.237 | 0.241 |
|  | 20-30 | 0.212 | 0.216 | 0.219 |
|  | 30-40 | 0.206 | 0.209 | 0.211 |
|  | 40-60 | 0.206 | 0.208 | 0.209 |
|  | 0-60 | 0.221 | 0.225 | 0.227 |
| S-30°-30%-3-2 | 0-10 | 0.323 | 0.336 | 0.350 |
|  | 10-20 | 0.277 | 0.285 | 0.291 |
|  | 20-30 | 0.268 | 0.274 | 0.278 |
|  | 30-40 | 0.270 | 0.276 | 0.280 |
|  | 40-60 | - | - | - |
|  | 0-60 | - | - | - |
| S-30°-30%-4-0 | 0-10 | 0.283 | 0.291 | 0.295 |
|  | 10-20 | 0.230 | 0.236 | 0.238 |
|  | 20-30 | 0.209 | 0.215 | 0.216 |
|  | 30-40 | 0.203 | 0.208 | 0.209 |
|  | 40-60 | 0.201 | 0.205 | 0.207 |
|  | 0-60 | 0.218 | 0.222 | 0.225 |
| P-00°-00%-0-0 | 0–20 | 1.588 | 1.879 | 1.879 |
|  |  |  |  |  |
|  | 20–40 | 0.667 | 0.693 | 0.693 |
|  |  |  |  |  |
|  | 40-60 | 0.556 | 0.556 | 0.565 |
|  | 0-60 | 1.042 | 1.044 | 1.049 |

**Annex Table 2 Median and IQR of the elastic moduli of all ranges and tested structures for the fast deformation speed**

Lower inter quartile range (IQR), Median and upper IQR of the elastic moduli for the different sample structures for the strain ranges 0-10%, 10-20%, 20-30%, 30-40% and 40-60% for the fast deformation and relaxation speed of 500 and 250 mm/min, respectively a strain rate of ~30 and ~15 %/s. Results of Agilus30Clear-Along[4] reprinted as reference for pure Agilus, denoted P-00°-00%-0-0.
